# Knowledge, attitudes and practices of infection control among staff working with learners with spina bifida in special schools in two South African provinces: A cross-sectional study

**DOI:** 10.4102/ajod.v15i0.1905

**Published:** 2026-05-12

**Authors:** Sasavona R. Mashamba, Saajida Mahomed, Jacqueline M. van Wyk

**Affiliations:** 1 School of Medicine, College of Health Sciences, University of KwaZulu-Natal, Durban, South Africa; 2 Department of Education, Tshilidzini Special School, Thohoyandou, South Africa; 3 Department of Health Science Education, Faculty of Health Sciences, University of Cape Town, Cape Town, South Africa

**Keywords:** spina bifida, infection control, knowledge, attitudes, practices

## Abstract

**Background:**

Infection associated with spina bifida is a common risk that often interferes with regular school attendance.

**Objective:**

This study aimed to conduct a situational analysis of infection control in South African special schools catering to learners with spina bifida, and to assess the knowledge, attitudes and practices of infection control among the school staff.

**Method:**

This was a cross-sectional study using semi-structured and structured questionnaires administered to the school principals and staff, respectively. A total of nine schools participated in the study, with 121 staff completing the questionnaire.

**Results:**

All nine schools reported a higher number of educators than allied health professionals, health professionals and house mothers. Low levels of knowledge about infection control were reported across all staff. A large proportion of educators (> 60%) were unaware of infection control practices. A small proportion across staff categories reported having a bowel management programme. Staff reported poor attitudes toward infection control; only 14.3% of educators, 15.0% of allied health staff, 13.3% of health staff and 6.3% of house mothers reported being trained to work with learners with spina bifida.

**Conclusion:**

The findings reveal important gaps in schools with regard to infection control for learners with spina bifida, and there is a need for training on infection control for all staff working in these settings.

**Contribution:**

The research contributes to the development of training and policy recommendations aimed at improving infection control for learners with spina bifida.

## Introduction

Spina bifida (SB) is the most common birth defect affecting the central nervous system and is often characterised as the most complex defect compatible with survival (Liptak & El Samra 2010).

Learners born with SB are at risk for hydrocephalus, leg weakness, paralysis, sensory loss and incontinence of the bladder and bowel (Mitchell et al. 2004). Advances made in reducing the under-5 child mortality globally have enabled the survival of more children with severe impairments. This achievement has ironically highlighted challenges in the health and education services that have not adequately kept pace with their needs (Miles 2006).

There are no recent statistics for South Africa, but a 1997 study showed an incidence of neural tube defects of 0.77 – 6.1 per 1000 live births, with a higher incidence in rural areas (Robertson et al. 1997). Lower incidence rates have been reported in urban regions such as Cape Town, with 1.3 per 1000 births (Buccimazza et al. 1994), and Johannesburg, with 1.18 per 1000 births (Kromberg & Jenkins 1982), compared to 3.5 per 1000 births in Limpopo province, a largely rural province (Ubbink et al. 1999).

Starting school is an important milestone for learners to develop greater responsibility and become more independent. Developing such independence can, however, be challenging for learners with spina bifida (LSB) (Sandler 2004), and many countries have educational policies that guide the support required for LSB. In Europe, LSB are often placed in mainstream schools or grouped in classes with learners with orthopaedic conditions (Lollar 2001). However, the policy of the National Department of Education on inclusivity does not make special provisions for LSB.

Similarly, in India, LSB access mainstream schools depending on the availability of family support for physical and toileting needs (Governey, Culligan & Leonard 2014). In South Africa, LSB are placed in special schools or classes or excluded from any educational opportunity because of the severity of their disability (Mashamba, Mahomed & Van Wyk 2025; Office of the Deputy President 1997). Learners with spina bifida in low-middle-income countries experience difficulties both in accessing school facilities and in moving around in their classrooms, while schools with boarding facilities that potentially could accommodate such learners lacked access to bathrooms, toilets, water or wheelchair access (Bannink, Idro & Van Hove 2016; Mathew 2006).

Bladder and bowel problems are present in more than 90% of learners with meningocele, regardless of the level of the lesion (Uehara 2002). Infection of the urinary tract may result from bladder paralysis, and wound infection can result from the lack of protective pain sensation (Swaroop & Dias 2009). Depending on the functional neurological level, LSB may have decreased or absent skin sensation, leaving them vulnerable to wound formation (Ottolini et al. 2013). The high level of care to manage LSB results in large numbers of learners having to travel great distances daily from their homes to attend highly specialised schools (Dhanook 2017). Schools offering specialised care for learners with severe and profound intellectual disabilities are scarce, have limited space, and are often very expensive (Mental Health Federation 2016). As a result, many parents are unable to find placement for their children or are unable to afford the fees, and the children are not able to receive a formal education (Mental Health Federation 2016). In South Africa, the limited availability and capacity of schools, particularly in rural areas, do not facilitate the integration of LSB into the mainstream and have led to many learners being excluded from educational opportunities (Office of the Deputy President 1997).

Schools that accommodate LSB must ensure that learners are managed in a manner that reduces their risk of acquiring infections. Effective infection control in schools is essential to supporting uninterrupted learning and minimising stigma. In this study, infection control referred to measures aimed at preventing infections among LSB, including condition-specific risks such as wound infection, pressure sores and shunt infections. General infection prevention practices, including hand hygiene and Coronavirus disease 2019 (COVID-19) precautions, were considered contextual factors that support overall learner safety but were not the primary focus of the study. There is relatively no information available on how schools in the African context support LSB concerning infections (Mashamba, Mahomed & Van Wyk 2024).

The objectives of this study were to conduct a situational analysis of infection control for LSB in South African special schools, and to assess the knowledge, attitudes, and practices of infection control among staff employed at these schools.

## Research methods and design

### Study design and setting

This was a cross-sectional study conducted in special schools accommodating LSB in two provinces of South Africa, that is, the Limpopo and Gauteng provinces. The schools were classified as public schools for learners with special needs (LSN). The 2005 policy statement of the Department of Basic Education defines a school for LSN as a resource to deliver education to learners requiring high-intensity education and other support on either a full-time or a part-time basis (Department of Education 2005). The Limpopo province is one of the poorest provinces in South Africa (Young 2020) and comprises a predominantly rural population of approximately 6 million people (Statistics South Africa 2020). Gauteng province is the most populous and has a population of approximately 16 million people (Statistics South Africa 2019). Gauteng province is considered the financial hub of the African continent; it is highly urbanised and considered the wealthiest province in South Africa (Statistics South Africa 2006). The choice of Gauteng province and Limpopo province was because of convenience for the principal investigator, and also to provide some insight into the state of infection control in special schools that represent a rural and an urban province.

### Participants and sampling

Limpopo province has 35 special schools for LSN, of which three accommodate learners with physical disabilities. Gauteng province has 120 special schools for LSN. For this study, 28 special schools accommodating learners with physical disabilities were purposively selected, including 25 from Gauteng province and three from Limpopo province.

The study population comprised principals and staff members working directly with LSB in selected special schools. Staff categories included educators, nurses, health promoters, physiotherapists, psychologists, speech therapists, occupational therapists, social workers and house mothers. In this context, a house mother referred to a staff member responsible for the care of LSB residing in school boarding facilities during the academic term. The inclusion criteria were school principals from the schools accommodating LSB, and staff in any of the aforementioned categories who were involved in managing LSB.

An email was sent to the principals of each of the 28 identified special schools informing them of the aim of the study and inviting their participation. Principals who consented to participate were asked to inform staff working with LSB about the study. Staff who expressed interest in participating agreed to have their contact details shared with the principal investigator (PI).

Of the 28 schools initially selected, six were excluded as a result of the absence of enrolled LSB. Of the remaining 22 schools, 13 did not provide consent to participate. In line with ethical requirements, no further data were collected from these schools, and their identifying information was not disclosed.

The sample was secured from the nine schools, representing the accessible portion of the population at the time of data collection. The principals of these nine schools and a total of 121 staff members consented to participate. The final sample size was therefore determined by institutional access and the willingness of eligible participants. The use of consent-driven sampling is widely accepted in educational and organisational research, particularly in studies involving defined professional groups and institution-based populations (Lohr 2019). In such contexts, the emphasis is placed on obtaining information-rich data from participants with direct relevance to the study objectives. Although the achieved sample size was limited to 121 participants, this number is consistent with widely cited methodological guidelines for exploratory and descriptive survey research, which indicate that samples of 100–200 participants are adequate for stable estimates and meaningful analysis (Israel 1992).

To address the potential gatekeeping role of school principals, it was emphasised that their role was limited to granting institutional access and circulating study information. Principals were not informed about the identities of the staff members who elected to participate, and no individual responses were shared with principals or school management.

Recruitment was slower than anticipated because of delayed responses from some schools. In certain cases, publicly listed email addresses were outdated or non-functional. Follow-up reminder emails were sent, and alternative contact details were sought to facilitate communication.

### Data collection

The questionnaires were developed by the PI, guided by existing literature and established infection control standards. To enhance content validity, the questionnaire was reviewed by a subject matter expert in SB. A pre-test was conducted with a small group of school staff working at a school accommodating LSB to assess clarity, relevance, and comprehension, and to refine the wording prior to full-scale data collection. The school selected for the pre-test was not part of the nine schools included in the main study.

The situational analysis was conducted using a structured questionnaire of closed and open-ended questions in English that was emailed to the school principals. The questionnaire elicited basic information relating to the number and age range of learners enrolled at the school, the number of LSB, the type of accommodation available for LSB, the number and category of staff employed and staff training requirements. In addition, the age and number of years of experience of the principals were obtained.

A closed-ended questionnaire in English was used to assess knowledge, attitudes and practices of infection control among members of staff. Section one elicited demographic variables and years of experience in special education settings. Section two explored participants’ knowledge of infection control. The questionnaires included information that clean intermittent catheterisation is a procedure to empty a bladder for LSB, heels, ankles, hips and elbows are prone to pressure sores on LSB, absent skin sensation in the trunk and buttocks can cause wound formation, headache, nausea or vomiting are the symptoms of shunt infection, coronavirus is transmitted through direct contact with respiratory droplets of an infected person and fever, cough, sore throat, headaches and difficulty in breathing are the symptoms of coronavirus.

Section three explored the participants’ attitudes toward infection control for LSB. Questionnaires included information about the staff’s level of attitude on the risk of infection in LSB, infection control is an important aspect in the management of LSB, the importance of infection control was emphasised in the schools, being comfortable assisting LSB affected with infections, staff were well trained to work with LSB and to prevent the spread of coronavirus on LSB.

Section four explored their practices of infection control towards LSB. The section included questionnaires on LSB were checked daily for pressure sores, burns and other injuries, LSB washed their hands frequently with soap and water, LSB were assisted to do wheelchair push-ups every 20–30 min, schools had bladder and bowel management programme, bladder and bowel management programme were practised daily at a scheduled time, schools were having catheterisation programme, schools were providing diapers for LSB with urine or bowel leakage, and learners with shunts were prevented from participating in contact sports.

With references to the section on knowledge and attitudes, they were asked to indicate their degree of agreement on each statement by using a 5-point Likert scale where 1 = strongly agree, 2 = agree, 3 = strongly disagree, 4 = disagree and 5 = neutral. Participants were asked to respond either by using ‘Yes’, ‘No’, or ‘I don’t know’ on statements relating to practices. The PI contacted each staff member either telephonically or via email, and questionnaires were emailed to those who had chosen to complete the questionnaire via email. All the completed questionnaires were returned to the PI. Data was collected from August 2020 to May 2021.

### Data analysis

Data from the questionnaires were entered into a Microsoft Excel spreadsheet. For data analysis, study participants were categorised into four groups based on their roles: educators, allied health (physiotherapists, speech therapists, occupational therapists and social workers), health staff (professional nurses, and auxiliary nurses), and house mothers. These groupings allowed for comparison of knowledge, attitudes and practices across different types of staff working with LSB.

For the 11 items assessing knowledge, 1 point was given for every correct answer. The six items assessing attitude were measured using a five-point Likert scale and scored as follows: strongly agree = 5, agree = 4, neutral = 3, disagree = 2 and strongly disagree = 1. Higher scores indicated more positive attitudes toward infection control. The responses were collapsed into three main categories: agree (combining strongly agree and agree) and disagree (combining disagree and strongly disagree) and neutral. The 13 practice items were measured using binary response options: Yes = 1 and No or I don’t know = 0. Percentages were then calculated and presented in tables to summarise participants’ responses.

Descriptive statistics, including frequencies, percentages, means and standard deviations, were calculated to summarise knowledge, attitudes and practices scores, demographic characteristics and school-level situational data. Spearman’s rank correlation was used to assess the association between knowledge, attitude and practice scores. A *p*-value < 0.05 was considered statistically significant.

### Ethical considerations

Ethical approval for the study was obtained from the University of KwaZulu-Natal Biomedical Research Ethics Committee (BREC/00000854/2019). Permission was obtained from the Gauteng and Limpopo Provincial Departments of Education and the principals of each of the schools to conduct the study. As a result of the COVID-19 pandemic and associated restrictions, in-person contact between the researcher and participants was minimised. As a result, informed consent was obtained and recorded telephonically prior to participation.

The University of KwaZulu-Natal Biomedical Research Ethics Committee approved a COVID-19 related amendment to the study protocol, permitting electronic data collection methods. Participants were informed of the study’s purpose, procedures, and their right to withdraw at any time. Participant anonymity was maintained by the removal of identifying information. Data were stored securely in digital files protected by a password and in locked physical storage, with access restricted to members of the research team only.

## Results

### Schools accommodating learners with spina bifida

Nine schools accommodating LSB were included: Six primary schools (Grades R–7), two combined schools (Grades R–12), and one junior primary school (Grades R–5). The total number of learners per school ranged from 184 to 389, with the number of LSB ranging from 1 to 25. Learners’ ages ranged from 5 years to 25 years (Table 1).

**TABLE 1:** Profile of schools accommodating learners with spina bifida in Gauteng and Limpopo provinces, 2021.

Province	Type of school	Number of learners enrolled	Age range of learners enrolled (years)	Number of LSB	Types and the number of professional staff	Leaner teacher ratio
Gauteng	Primary school	389	5–21	1	1 Professional nurse 1 Social worker 2 Occupational therapists 1 Counsellor	1:16
	Primary school	124	5–15	10	2 Professional nurse 2 Physiotherapist 2 Speech therapist 2 Psychologist 2 Social workers 2 Occupational therapists	1:9
	Primary school	365	5–18	3	1 Professional nurse 1 Physiotherapist 1 Speech therapist 1 Social worker 3 Occupational therapists	1:11
	Junior primary school	304	5–21	1	1 Professional nurse 1 Social worker 2 Occupational therapists	1:11
	Combined school	380	5–25	20	1 Professional nurse 4 Physiotherapist 1 Speech therapist 2 Social workers 4 Occupational therapists 1 Audio therapist	1:15
	Primary school	370	5–18	1	2 Professional nurse 2 Physiotherapist 5 Occupational therapists	1:10
Limpopo	Combined school	264	5–25	25	5 Auxiliary nurses 1 Social worker	1:9
	Primary school	334	5–16	15	1 Professional nurse 2 Auxiliary nurses 1 Physiotherapist 1 Social worker	1:10
	Primary school	184	5–18	10	1 Professional nurse	1:15

LSB, learners with spina bifida.

### Physical infrastructure of the schools for learners with spina bifida

All nine schools (100%) reported having running water, flushable toilets and wheelchair-accessible bathrooms equipped with basins, soap, and paper towels. Four schools (44%) had boarding facilities. None of the schools had separate classrooms for LSB.

### Human resources of the schools for learners with spina bifida

The average years of experience as a school principal were seven years, with a range of 10 years to 29 years. Four (44%) schools did not have a house mother, and five (56%) schools reported having a house mother. The schools employed various professional staff (refer to Table 1). Four schools (44%) did not employ a house mother, while five schools (56%) did. The minimum qualifications varied: one school (11%) required Grade 10, two schools (22%) required Grade 12, two schools (22%) required a home-based care qualification and four schools (44%) did not require any specific qualification.

### Training for staff and principals

Training related to infection control and the management of LSB varied across schools. Only one (11%) principal reported that house mothers received school-based infection control training. In contrast, six (67%) principals indicated that educators had received training, although the format and focus differed between institutions. Reported training topics included augmentative and alternative communication (AAC), workshops to increase awareness of SB, inclusive education workshops, infection prevention and support for LSN and formal qualifications such as an Advanced Certificate in Education for LSN offered by universities. Training for educators on infection control and management of LSB was provided by the Provincial Department of Education.

Five (56%) principals reported that they attended training addressing the needs of LSN and the management of infection among LSB. This training primarily focused on managing educators, support staff and broader institutional responsibilities. Delivery methods included workshop-based sessions and formal academic programmes, such as higher degrees or Advanced Certificates in Education. Training for principals was provided by the Provincial Department of Education and universities.

Regarding the frequency of training for the principals, nine schools offered training very often. A similar pattern was observed for educators, often reported in seven schools. Notably, six of the nine schools did not offer infection control training specifically for LSB. Training was extended to parents of LSB in three schools and to non-teaching staff in one school. All nine schools reported receiving COVID-19-related training provided by the respective Provincial Departments of Education.

### Knowledge, attitudes and practices of staff managing learners with spina bifida

A total of 121 staff members participated. The majority were female (*n* = 91, 75.2%), with males constituting 24.8% (*n* = 30). Most participants worked in primary schools (67.8%), followed by combined schools (21.5%) and junior secondary schools (10.7%). Participants’ ages were distributed as follows: 28–39 years (11.6%), 40–49 years (46.3%) and 50+ years (42.1%). The largest occupational group was educators (57.9%), followed by house mothers (13.2%), nurses (12.4%), occupational therapists (7.4%), physiotherapists (4.1%), social workers (3.3%) and speech therapists (1.7%). Years of experience were distributed as 0–9 years (32.2%), 10–19 years (44.6%) and 20+ years (23.1%). Geographically, 60.3% of participants were from Gauteng province and 39.7% from Limpopo province.

Table 2, Table 3, and Table 4 present responses about knowledge, attitudes and practices of infection control among staff working with LSB in Gauteng province and Limpopo province.

**TABLE 2:** Knowledge of infection control among staff by occupational group.

Statements	Educators (*n* = 70)		Allied health (*n* = 20)		Health (*n* = 15)		House mothers (*n* = 16)	
	*n*	%	*n*	%	*n*	%	*n*	%
Clean intermittent catheterisation is a procedure used to empty the bladder.	27	38.6	6	30.0	6	40.0	4	25.0
A catheter is used to drain urine.	29	41.4	9	45.0	7	46.7	5	31.3
Adding extra fibre to the diet of a learner with spina bifida can help improve the regularity of bowel movements.	18	25.7	7	35.0	5	33.3	2	12.5
Heels are prone to pressure sores.	20	28.6	6	30.0	6	40.0	4	25.0
Ankles are prone to pressure sores.	11	15.7	5	25.0	4	26.7	1	6.3
Hips are prone to pressure sores.	12	17.1	5	25.0	4	26.7	1	6.3
Elbows are prone to pressure sores.	10	14.3	5	25.0	2	13.3	1	6.3
Absent skin sensations in the trunk and buttocks can cause wound formation.	28	40.0	5	25.0	3	20.0	0	0.0
Headache, nausea or vomiting are the symptoms of shunt infection.	21	30.0	9	45.0	7	46.7	4	25.0
Coronavirus is transmitted through direct contact with respiratory droplets of an infected person generated through coughing and sneezing.	57	81.4	18	90.0	12	80.0	14	87.5
Fever, cough, sore throat, headaches and difficulty in breathing are the symptoms of COVID-19.	62	88.6	20	100.0	14	93.3	15	93.8

Note: Percentages calculated within each occupational group. Values represent participants selecting ‘agree’ or ‘strongly agree’.

COVID-19, coronavirus disease 2019.

**TABLE 3:** Attitude of infection control among staff by occupational group.

Statement	Educators (*n* = 70)		Allied health (*n* = 20)		Health (*n* = 15)		House mothers (*n* = 16)	
	*n*	%	*n*	%	*n*	%	*n*	%
I’m concerned about the risk of infections in LSB in my school.	27	38.6	8	40.0	6	40.0	9	56.3
Infection control is an important aspect of the management of LSB.	30	42.9	9	45.0	8	53.3	8	50.0
The importance of infection control for spina bifida is emphasised in my school.	23	32.9	5	25.0	2	13.3	2	12.5
I’m comfortable assisting LSB affected with infections.	20	28.6	9	45.0	10	66.7	2	12.5
I am well-trained to work with LSB.	10	14.3	3	15.0	2	13.3	1	6.3
I am well-trained to prevent the spread of coronavirus on LSB.	16	22.9	5	25.0	3	20.0	3	18.8

Note: Percentages calculated within each occupational group. Values represent participants selecting ‘agree’ or ‘strongly agree’.

LSB, learners with spina bifida.

**TABLE 4:** Practices of infection control among staff by occupational group.

Statement	Educators (*n* = 70)		Allied health (*n* = 20)		Health (*n* = 15)		House mothers (*n* = 16)	
	*n*	%	*n*	%	*n*	%	*n*	%
LSB are checked every day for pressure sores.	14	20.0	6	30.0	9	60.0	6	37.5
LSB are checked every day for burns.	11	15.7	5	25.0	9	60.0	5	31.3
LSB are checked every day for other injuries.	12	17.1	5	25.0	9	60.0	5	31.3
LSB are assisted to wash their hands frequently with soap and water.	25	35.7	7	35.0	12	80.0	6	37.5
LSB are assisted to do wheelchair push-ups every 20–30 min.	7	10.0	2	10.0	4	26.7	1	6.3
My school has a bladder management programme for LSB.	8	11.4	2	10.0	6	40.0	1	6.3
A bladder management programme for LSB is practised every day at a scheduled time.	5	7.1	1	5.0	4	26.7	1	6.3
My school has a bowel management programme for LSB.	6	8.6	2	10.0	4	26.7	1	6.3
A bowel management programme for LSB is practised every day at a scheduled time.	4	5.7	1	5.0	4	26.7	1	6.3
There is a catheterisation programme at my school.	4	5.7	1	5.0	3	20.0	0	0.0
The school has a scheduled time for catheterisation.	2	2.9	1	5.0	3	20.0	0	0.0
The school provides diapers for LSB who have urine or bowel leakage.	1	1.4	1	5.0	2	13.3	0	0.0
Learners with shunts are prevented from participating in contact sports.	5	7.1	2	10.0	3	20.0	1	6.3

Note: Percentages calculated within each occupational group. Values represent participants reporting ‘yes’.

LSB, learners with spina bifida.

The mean knowledge score among staff was 3.80 (standard deviation [s.d.] = 2.56), indicating generally low to moderate knowledge. According to the knowledge statements, only a minority of health staff recognised that clean intermittent catheterisation is a procedure to empty a bladder. Similarly, a comparable proportion correctly identified the heels as being prone to pressure sores (Table 2).

The staff who agreed that ankles are prone to pressure sores were eleven (15.7%) educators and five (25.0%) allied health. Similarly, low levels of knowledge were reported across all staff categories about hips and elbows being prone to pressure sores. Most health staff agreed that the absence of skin sensation in the trunk and buttocks can cause wound formation, and most staff reported knowing how the coronavirus was transmitted and the symptoms thereof.

The mean attitude score was 17.21 (s.d. = 3.90), ranging from 11 to 30, reflecting moderately positive attitudes. A larger proportion of house mothers agreed that they were concerned about the risk of infection in LSB compared to other staff categories. The proportion of staff who agreed that infection control was an important aspect in the management of LSB varied across the staffing categories (Table 3). Ten health staff (66.7%) agreed that they were comfortable assisting LSB affected with infections. Very few staff agreed that they were well-trained to work with LSB.

The mean practice score was 1.64 (s.d.= 2.27), with a range of 0–12, indicating very low adherence to recommended practices. Most health staff agreed that LSB were checked daily for pressure sores, burns, and injuries. The proportion of staff who agreed that LSB were assisted with washing their hands frequently with soap and water varied from 25 educators (35.7%), 12 health staff (80.0%), seven allied health (35.0%) and six house mothers (37.5). Eight educators (11.4%), two allied health professionals (10.0%), six health staff (40.0%) and one house mother (6.3%) indicated that their school had a bladder management programme. Only a small proportion of staff across the different categories of occupation reported that the programme was practised daily at a scheduled time. A small proportion of participants across staff categories reported that their schools had a bowel management programme, and even fewer indicated that the programme was practised daily at a scheduled time. Similarly, fewer categories of staff reported that catheterisation programmes exist at their schools. Very few participants reported that diapers were provided for learners experiencing urine or bowel leakage.

### Relationship between knowledge, practice and attitude scores

Spearman’s rank order correlation analysis demonstrated statistically significant positive correlations among all three domains (p < 0.001). Knowledge was moderately correlated with practice (ρ = 0.423, p < 0.001) and attitude (ρ = 0.521, *p* < 0.001). Attitude was also moderately correlated with practice (ρ = 0.434, *p* < 0.001).

## Discussion

This study provides a descriptive overview of the knowledge, attitudes and practices related to infection control among staff working with LSB. Understanding the broader context in which LSB access education is important because workplace factors influence how staff implement infection control practices. The number of LSB attending special schools may reflect limited access to such facilities, particularly in rural areas where transport and affordability are barriers. Although South Africa has an inclusive education policy, very few public mainstream schools can accommodate LSB who require significant medical or physical support (Ramsundhar & Donald 2014). Learners with severe SB symptoms may be kept out of school entirely if they cannot access special schools (Ramsundhar & Donald 2014).

Three of the four schools with boarding facilities were in Limpopo province, likely reflecting the long distances learners in rural provinces must travel. One school in Gauteng province had a house mother but no boarding facilities. While principals of schools accommodating LSB had, on average, over 20 years of experience, their training focused primarily on general school management, with few reporting training in infection control for LSB.

Schools accommodating LSB reported higher numbers of educators than allied health staff, nurses or house mothers. This contrasts with the South African Department of Education policy, which allows non-teaching and non-professional staff in special schools to exceed teaching staff numbers to meet learners’ physical needs, especially in the foundational phase (ed. Department of Basic Education 2014). The staffing patterns observed in this study may limit the consistent provision of infection control support within these school settings. Schools in Gauteng province generally had more allied health staff than those in Limpopo province, reflecting provincial disparities.

This study found poor knowledge of infection control among educators, allied health and house mothers. This is similar to a study in the United States on teacher knowledge and confidence to manage children with chronic medical conditions, where less than 10% of teachers reported being well-informed about SB (Nabors et al. 2008). As expected, health staff had better knowledge of infection control compared to all other categories of staff. The majority of staff were knowledgeable about the transmission of COVID-19. This finding was not surprising since the study was conducted during a period of the pandemic when there were many public messages on COVID-19 and the Department of Education provided training on COVID-19.

Limited knowledge of infection control measures may increase the risk of infection associated with the care and management of LSB in the school environment. These findings suggest the need for structured training initiatives aimed at improving staff knowledge. School principals and administrators should ensure that infection control education forms part of mandatory induction programmes for newly appointed staff. Continuous professional development opportunities may further strengthen staff knowledge and ensure that infection control policies are clearly understood and consistently applied.

Staff reported not being well-trained to work with LSB. Previous studies suggest that collaborative training initiatives may support educators in developing a better understanding of the needs of LSB and approaches to supporting them in educational settings (Gaintza, Ozerinjauregi & Aróstegui 2018). Other studies have identified the medical team for LSB, parents, and school nurses as potential training and information sources for teachers (Nabors et al. 2008). The findings of this study suggest the need for improved and regular information sessions for all staff working at schools for LSB.

The majority of staff reported that they were not concerned about the risk of infection among LSB and that they were uncomfortable in assisting LSB affected with infections. Staff must be provided with knowledge and skills about infection control to improve their attitude and confidence when supporting LSB. This finding is similar to a study where teachers’ attitudes were linked to their lack of knowledge (Gaintza et al. 2018).

We also found poor infection control practices, despite the report from the principals affirming that educators were trained to respond to the needs of LSB. The large proportion of educators who were unaware of practices in their schools regarding infection control, bladder management programmes and or schedules for learners is a cause for concern. The majority of staff reported that their schools do not provide diapers for LSB who have urine or bowel leakage. Leakage and the subsequent odour may cause stigmatisation and isolation (Toefy 2014). A study in Australia recommended developing an individual care plan and continence care plan, which can be carried out by a health professional, who will provide instruction about the individual management of SB in the school (Department for Education 2019).

The study findings on poor infection control practices highlight the importance of strengthening practical training and ongoing supervision within the school environment. School management teams should consider implementing routine monitoring of infection control practices to ensure adherence to established guidelines. Collaboration between school administrators, healthcare professionals and parents may further support the effective implementation of infection prevention measures in special school settings.

Future research should focus on generating more comprehensive evidence on infection risks among LSN and the preparedness of school staff to manage these risks. Further studies could also explore specific knowledge and practice gaps across different staff categories and evaluate the effectiveness of targeted training programmes aimed at improving infection control practices in special school environments.

The study found significant positive correlations among knowledge, practice and attitude, indicating that higher knowledge is associated with more positive attitudes and better infection control practices. These results align with previous studies showing that knowledge influences both attitudes and practice (Kumar et al. 2021). Further research is required to assess the role of personal and environmental factors that may influence knowledge, attitude and practices.

While this study focused on LSB, the findings have implications for infection control for all learners, particularly those with disabilities who may be more vulnerable to infections. Ensuring staff have adequate knowledge, positive attitudes, and effective practices supports inclusive education, health equity and safer learning environments (Centers for Disease Control and Prevention 2024; World Health Organization 2020)

### Limitations of the study

This study has several limitations that should be considered when interpreting the findings. Firstly, it was conducted in only two provinces in South Africa (Gauteng province and Limpopo province), which limits the generalisability of the findings. However, these provinces reflect disparities in urban and rural settings, and schools in other provinces likely face similar challenges regarding infrastructure, staffing and infection control.

Secondly, the study was conducted as a cross-sectional survey and did not include multiple sources of information typically used in a full situational analysis. Therefore, the findings reflect staff-reported knowledge, attitudes and practices regarding infection control and may not capture all aspects of the school context. Future studies incorporating multiple data sources could provide a more comprehensive situational analysis and inform policy and practice for inclusive and safe school environments.

Thirdly, practices were self-reported rather than directly observed. This raises the possibility of information bias, where participants may overestimate adherence or report socially desirable behaviours. Fourthly, the response rate from schools was low, with less than 50% of eligible schools consenting to participate, which may limit the generalisability of the findings. Schools that declined participation may differ systematically from those that participated, introducing potential selection bias.

A further limitation relates to the use of school principals as gatekeepers. While necessary to gain access, this approach may have influenced participation. Principals could have selectively facilitated access to certain staff members, introducing selection bias.

## Conclusion

The findings of this study reveal that special schools have deficiencies in providing adequate infection control to support LSB in the South African setting. Staff also demonstrated poor knowledge, attitudes and practices on how to manage infection control amongst LSB. These findings suggest that LSB may be at an unnecessarily higher risk of acquiring preventable infections. Improving the knowledge of infection control among all staff is likely to encourage better attitudes and practices of infection control within the school. Future research in this underexplored area is needed to determine whether strengthening infection control practices in schools leads to improved health outcomes for LSB, and to identify the specific requirements in terms of human resources, supplies and medical equipment necessary to ensure effective infection control in these settings.

## References

[CIT0001] Bannink, F., Idro, R. & Van Hove, G., 2016, ‘Teachers’ and parents’ perspectives on inclusive education for children with spina bifida in Uganda’, *Journal of Childhood and Developmental Disorders* 2(2), 1–8. 10.4172/2472-1786.100026

[CIT0002] Buccimazza, S.S., Molteno, C.D., Dunne, T.T. & Viljoen, D.L., 1994, ‘Prevalence of neural tube defects in Cape Town, South Africa’, *Teratology* 50, 194–199. 10.1002/tera.1420500304 7871483

[CIT0003] Centers for Disease Control and Prevention (CDC), 2024, *Preventing spread of infections in K-12 schools*, Atlanta, Georgia.

[CIT0004] Department for Education, S.A., 2019, *Spina bifida and hydrocephalus health support for children and young people*, Department for Education, Adelaide, viewed 25 June 2024, from https://www.education.sa.gov.au/schools-and-educators/health-safety-and-wellbeing/specific-health-conditions-and-needs/spina-bifida-and-hydrocephalus-healt-support-children-and-young-people.

[CIT0005] Department of Basic Education, 2014, *Guidelines to ensure quality education and support in special schools and special school resource centres to support inclusive education*, Department of Basic Education, Pretoria.

[CIT0006] Department of Education, 2005, *Guidelines to ensure quality education and support in special schools and special school resource centres*, Department of Education, Pretoria.

[CIT0007] DhanooK, I, 2017, *Special needs schools lack support staff, The Post (IOL)*, Independent Media, Johannesburg, viewed 17 May 2025, from https://www.iol.co.za/thepost/news/2017-12-08-specal-needs-schoolslack-support-staff/

[CIT0008] Gaintza, Z., Ozerinjauregi, N. & Aróstegui, I., 2018, ‘Educational inclusion of students with rare diseases: Schooling students with spina bifida’, *British Journal of Learning Disabilities* 46, 250–257. 10.1111/bld.12246

[CIT0009] Governey, S., Culligan, E. & Leonard, J., 2014, *The health and therapy needs of children with spina bifida in Ireland*, Temple Street Children’s University hospital, Dublin.

[CIT0010] Israel, G.D., 1992, *Determining sample size*, University of Florida, Institute of Food and Agricultural Sciences, Gainesville, FL.

[CIT0011] Kromberg, J.G. & Jenkins, T., 1982, ‘Common birth defects in South African Blacks’, *South African Medical Association* 62, 599–602. 6750816

[CIT0012] Kumar, N., Elango, S., Chatterjee, S., Das, N. & Singh, R., 2021, ‘Knowledge, attitudes, and practices among HIV/Leishmaniasis co-infected patients in Bihar, India’, *PLoS One* 16, e0261933. 10.1371/journal.pone.0261933 34962969 PMC8714085

[CIT0013] Liptak, G.S. & El Samra, A., 2010, ‘Optimizing health care for children with spina bifida’, *Developmental Disabilities Research Reviews* 16, 66–75. 10.1002/ddrr.91 20419773

[CIT0014] Lohr, S.L., 2019, *Sampling: Design and analysis*, CRS Press, Boca Raton, FL.

[CIT0015] Lollar, D.J., 2001, *Learning among children with spina bifida*, Spina Bifida Association, Washington, DC.

[CIT0016] Mashamba, S.R., Mahomed, S. & Van Wyk, J.M., 2024, ‘Infection control in schools for learners with spina bifida: A scoping review’, *African Journal of Disability* 13, 1394. 10.4102/ajod.v13i0.1394 39229351 PMC11369540

[CIT0017] Mashamba, S.R., Mahomed, S. & Van Wyk, J.M., 2025, ‘Challenges faced by staff managing learners with spina bifida in South African special schools’, *Health SA Gesondheid* 30, 2993. 10.4102/HSAG.v30i0.2993 41069553 PMC12505408

[CIT0018] Mathew, G, 2006, *Study to assess the compliance and selected factors affecting compliance to clean intermittent catheterisation (CIC) in children with spina bifida with a view to develop guidelines for home management in the Indian setting*, Unpublished MSc Nursing dissertation, Rajiv Gandhi University of Health Sciences (RGUHS), Bangalore.

[CIT0019] Mental Health Federation, S.A., 2016, *Who will look after my disabled child while I work*, News 24 (ed.), News 24, Cape Town.

[CIT0020] Miles, M., 2006, ‘Children with spina bifida and hydrocephalus in Africa: Can medical, family and community resources improve the life chances’, *Disability & Society* 17, 643–658. 10.1080/0968759022000010425

[CIT0021] Mitchell, L.E., Adzick, N.S., Melchionne, J., Pasquariello, P.S., Sutton, L.N. & Whitehead, A.S., 2004, ‘Spina bifida’, *Lancet* 364, 1885–1895. 10.1016/S0140-6736(04)17445-X 15555669

[CIT0022] Nabors, L.A., Little, S.G., Akin-Little, A. & Iobst, E.A., 2008, ‘Teacher knowledge of and confidence in meeting the needs of children with chronic condition’, *Psychology in the Schools* 45(3), 217–226. 10.1002/pits.20292

[CIT0023] Office of the Deputy President, 1997, *White Paper on an Integrated National Disability Strategy*, Office of the Deputy President, Pretoria.

[CIT0024] Ottolini, K., Harris, A.B., Amling, J.K., Kennelly, A.M., Phillips, L.A. & Tosi, L.L., 2013, ‘Wound care challenges in children and adults with spina bifida: An open-cohort study’, *Journal of Pediatric Rehabilitation Medicine* 6, 1–10. 10.3233/PRM-130231 23481886

[CIT0025] Ramsundhar, N. & Donald, K., 2014, ‘An approach to the developmental and cognitive profile of the child with spina bifida’, *South African Medical Journal* 104(3), 221. 10.7196/SAMJ.8048

[CIT0026] Robertson, H.-L., Steyn, N.P., Venter, P.A. & Christianson, A., 1997, ‘Neural tube defects and folic acid: A South African perspective’, *South African Medical Journal* 87(7), 928–931.

[CIT0027] Sandler, A., 2004, *Living with spina bifida: A guide for families and professionals*, University of North Carolina Press, Chapel Hill, North Carolina.

[CIT0028] Statistics South Africa, 2006, *Statistics in brief 2006*, Statistics South Africa, Pretoria.

[CIT0029] Statistics South Africa, 2019, *Mid-year population estimates 2019 (Statistical release P0302)*, Statistics South Africa, Pretoria.

[CIT0030] Statistics South Africa, 2020, *General household survey 2018 (Statistical release P0318)*, Statistics South Africa, Pretoria.

[CIT0031] Swaroop, V.T. & Dias, L., 2009, ‘Orthopedic management of spina bifida. Part I: hip, knee, and rotational deformities’, *Journal of Children’s Orthopaedics* 3, 441–449. 10.1007/s11832-009-0214-5 PMC2782071 19856195

[CIT0032] Toefy, Z., 2014, ‘Beyond the operating theatre: Long-term quality of life in spina bifida’, *South African Medical journal* 104, 317. 10.7196/SAMJ.8148

[CIT0033] Ubbink, J.B., Christianson, A., Bester, M.J., Van Allen, M.I., Venter, P.A., Delport, R. et al., 1999, ‘Folate status, homocysteine metabolism, and methylene tetrahydrofolate reductase genotype in rural South African blacks with a history of pregnancy complicated by neural tube defects’, *Metabolism* 48, 269–274. 10.1016/S0026-0495(99)90046-X 10024094

[CIT0034] Uehara, M., 2002, ‘Neural tube defects’, in L.G. Yamamoto et al. (eds.), *Case based pediatrics for medical students and residents*, Department of Pediatrics, University of Hawaii John A, Burns School of Medicine, Honolulu, HI, viewed 10 October 2023, from https://www.hawaii.edu/medicine/pediatrics/pedtext/s18c10.html.

[CIT0035] World Health Organization, 2020, *Infection preventon and control during health care when COVID-19 is suspected or confirmed*, World Health Organization, Geneva.

[CIT0036] Young, J., 2020, *Advantages of Limpopo province as business destination*, Global Africa Network, Limpopo.

